# Prevalence of schizophrenia spectrum and other psychotic disorders in problem gambling: A systematic review and meta-analysis

**DOI:** 10.1192/j.eurpsy.2024.1777

**Published:** 2024-09-25

**Authors:** Olivier Corbeil, Laurent Béchard, Élizabeth Anderson, Maxime Huot-Lavoie, Charles Desmeules, Lauryann Bachand, Sébastien Brodeur, Pierre-Hugues Carmichael, Christian Jacques, Marco Solmi, Michel Dorval, Isabelle Giroux, Marc-André Roy, Marie-France Demers

**Affiliations:** 1 Faculty of Pharmacy, Université Laval, Quebec, QC, Canada; 2 Quebec Mental Health University Institute, Quebec, QC, Canada; 3 CERVO Brain Research Centre, Quebec, QC, Canada; 4 School of Psychology, Université Laval, Quebec, QC, Canada; 5 Faculty of Medicine, Université Laval, Quebec, QC, Canada; 6 Institute of Psychiatry, Psychology and Neuroscience, King’s College London, London, UK; 7 Centre d’Excellence sur le Vieillissement de Québec, Quebec, QC, Canada; 8 Centre Québécois d’Excellence pour la Prévention et le Traitement du Jeu, Quebec, QC, Canada; 9 SCIENCES Lab, Department of Psychiatry, University of Ottawa, Ottawa, ON, Canada; 10 Department of Mental Health, The Ottawa Hospital, Regional Centre for the Treatment of Eating Disorders and On Track: The Champlain First Episode Psychosis Program, Ottawa, ON, Canada; 11 Ottawa Hospital Research Institute (OHRI), Clinical Epidemiology Program, University of Ottawa, Ottawa, ON, Canada; 12 Department of Child and Adolescent Psychiatry, Charité Universitätsmedizin, Berlin, Germany; 13 CHU de Québec – Université Laval Research Centre, Quebec, Canada

**Keywords:** gambling, meta-analysis, prevalence, psychosis, schizophrenia

## Abstract

**Background:**

High rates of psychiatric comorbidities have been found in people with problem gambling (PBG), including substance use, anxiety, and mood disorders. Psychotic disorders have received less attention, although this comorbidity is expected to have a significant impact on the course, consequences, and treatment of PBG. This review aimed to estimate the prevalence of psychotic disorders in PBG.

**Methods:**

Medline (Ovid), EMBASE, PsycINFO (Ovid), CINAHL, CENTRAL, Web of Science, and ProQuest were searched on November 1, 2023, without language restrictions. Studies involving people with PBG and reporting the prevalence of schizophrenia spectrum and other psychotic disorders were included. Risk of bias was assessed using the Joanna Briggs Institute critical appraisal checklist for systematic reviews of prevalence data. The pooled prevalence of psychotic disorders was calculated using a random effects generalized linear mixed model and presented with forest plots.

**Results:**

Of 1,271 records screened, 22 studies (*n* = 19,131) were included. The overall prevalence of psychotic disorders was 4.9% (95% CI, 3.6–6.5%, *I*^2^ = 88%). A lower prevalence was found in surveyed/recruited populations, compared with treatment-seeking individuals and register-based studies. No differences were found for factors such as treatment setting (inpatient/outpatient), diagnoses of psychotic disorders (schizophrenia only/other psychotic disorders), and assessment time frame (current/lifetime). The majority of included studies had a moderate risk of bias.

**Conclusions:**

These findings highlight the relevance of screening problem gamblers for schizophrenia spectrum and other psychotic disorders, as well as any other comorbid mental health conditions, given the significant impact such comorbidities can have on the recovery process.

## Introduction

A growing consensus in both research and clinical practice emphasizes the importance of adopting a transdiagnostic and holistic approach toward the care of people with mental health disorders [1, 2]. This approach goes beyond the treatment of the underlying primary psychiatric disorder to address the biopsychosocial factors that are central to recovery, surpassing mere symptom management. In light of this, a better understanding of the co-occurrence of conditions that may exacerbate the challenges faced by individuals becomes imperative [3–7].

Within the diverse spectrum of mental health disorders, problem gambling (PBG) has received increasing attention, particularly given the growing availability of various forms of gambling, including its increasingly widespread integration into online video games [8–10]. Since the publication of the fifth edition of the Diagnostic and Statistical Manual of Mental Disorders (DSM-5) in 2013, gambling disorder, which can be considered a more severe form of PBG, has been officially recognized as a behavioral addiction [11]. This is due, in part, to growing evidence that gambling disorder and PBG share many similarities with substance addiction [12]. Studies have also suggested a complex relationship between PBG and psychiatric comorbidities, including mood and anxiety disorders [13, 14].

High rates of psychiatric comorbidity have been reported in people with PBG. A recent nationwide registry study found that 77% of patients with gambling disorder (*n* = 2,099) had at least one co-occurring psychiatric diagnosis, with anxiety, affective, and substance use disorders being the most prevalent [15]. A previous meta-analysis focusing on treatment-seeking individuals with PBG mirrored these patterns, reporting prevalences of current and lifetime comorbid Axis I disorders at 75% [13]. Major depressive disorder (29.9, 95% confidence interval [CI], 20.5–41.3%, 17 studies), substance use disorders (22.2, 95% CI, 16.1–29.8%, 26 studies), and anxiety disorders (17.6, 95% CI, 10.8–27.3%, 15 studies) were identified as the most common current comorbid diagnoses [13]. In addition, the prevalence of comorbid personality disorders, particularly cluster B disorders, could be as high as 47.9% (95% CI, 29.8–66.7%, 15 studies) [16]. Importantly, these comorbid mental health disorders add on to the challenges people with PBG struggle with, further diminishing their quality of life and impeding their recovery, thereby highlighting the need for integrative treatment approaches [17].

While there is a relatively large body of research examining the comorbidity of PBG with various mental health conditions, psychotic disorders have received less attention. Yet, in addition to the significant burden in terms of reduced quality of life and premature mortality associated with psychotic disorders [18–20], there is evidence suggesting that their co-occurrence with PBG may lead to more severe gambling problems, including greater financial difficulties and an increased risk of homelessness and suicidality [21–27]. A recent meta-analysis published by our group, including 12 studies and 3,443 individuals, estimated the overall prevalence of PBG in people with psychotic disorders to be 8.7% [28]; in comparison, the prevalence of PBG in the general population worldwide is estimated to be 1.3% [29]. Although not directly compared, this suggests that PBG may be more common in people with psychotic disorders than in the general population. Conversely, at the time of publication of the most recent meta-analysis in 2015, only five studies had provided data on the prevalence of psychotic disorders among problem gamblers [13]. In the latter, the prevalence of psychotic disorders was estimated to be 4.7% in a relatively small sample of only 989 individuals with PBG, while psychotic disorders are estimated to affect approximately 1% of the global population [20, 30]. Since then, a substantial number of larger observational studies have been conducted [26, 31, 32], which may allow a more accurate estimate of the prevalence of psychotic disorders in people with PBG. This may also enable the investigation of variations due to methodological and population factors, which have not yet been done. This investigation is important because the increased prevalence of PBG in people with psychotic disorders found in our recent meta-analysis does not necessarily indicate that the prevalence of psychotic disorders in people with PBG is also increased compared to the corresponding prevalence in the general population, as these are two distinct populations with different vulnerability profiles. Therefore, the aim of this systematic review and meta-analysis was to estimate the prevalence of schizophrenia spectrum and other psychotic disorders in individuals with PBG and to examine potential variations due to factors such as population and treatment setting.

## Methods

The reporting of this study conforms to the standards of the Preferred Reporting Items for Systematic reviews and Meta-Analyses (PRISMA) 2020 statement [33]. This review was registered in the International Prospective Register of Systematic Reviews (PROSPERO) on June 30, 2023 (CRD42023428242).

### Search strategy and selection criteria

A comprehensive search strategy was developed with a health sciences librarian, and the following databases were searched on November 1, 2023: Medline (Ovid), EMBASE, PsycINFO (Ovid), CINAHL, Cochrane Central Register of Controlled Trials (CENTRAL), Web of Science, and ProQuest Dissertation and Thesis. The search strategy included a combination of terms related to gambling (e.g., “gambling” or “betting”) and psychotic disorders (e.g., “psychosis” or “schizophrenia”) in both controlled vocabulary and free text (Supplementary Table 1). Relevant peer-reviewed studies were also identified through a manual review of the reference lists of included studies and Google Scholar. There were no language restrictions.

Studies were included if they met the following inclusion criteria: 1) included a sample of individuals (of any age) with PBG (including but not limited to gambling disorder, and as classified by the authors based on DSM/ICD criteria or other clinically relevant definition); 2) reported on the prevalence of schizophrenia spectrum and other psychotic disorders (as classified by the authors based on DSM/ICD criteria), including non-affective (i.e., schizophrenia spectrum disorders) and psychotic mood disorders (i.e., bipolar disorder and depression with psychotic features); 3) observational (i.e., cohort, nested case-control, cross-sectional) and experimental (i.e., randomized and non-randomized trials) designs. There were no other exclusion criteria. If multiple articles reported data from the same study sample, only the article reporting the most detailed results (i.e., in terms of the data extracted for this review) or with the largest sample size was retained for inclusion.

After removing duplicate records, the principal investigator (O.C.) and an undergraduate psychology student (E.A.) independently screened titles/abstracts and then assessed relevant full-text articles for inclusion. Covidence systematic review software facilitated the screening process [34]. Discrepancies in judgment were thoroughly discussed, and consensus was reached or resolved by a third reviewer (L. Béchard).

### Data extraction

The extraction of data was carried out by the principal investigator (O.C.) and two undergraduate psychology students (E.A. and L. Bachand). The information extracted included: 1) study design (e.g., cross-sectional, cohort, case-control, prevalence/survey, randomized controlled trial); 2) country, further categorized according to the regional distribution established by the World Health Organization (WHO), including Africa, Eastern Mediterranean, Europe, Americas, South-East Asia, and Western Pacific, as well as according to the World Bank income classifications for 2022 (i.e., high-income or low- and middle-income); 3) age and sex or gender criteria for inclusion; 4) mean age of the population; 5) proportion of men and women; 6) proportion of White and African/Afro-American (as these ethnicities were expected to be the most represented in the studies conducted to date, based on a previous review [35]); 7) type of recruitment (i.e., treatment-seeking individuals, surveyed/recruited, register-based, other); 8) treatment setting (i.e., inpatient, outpatient, mixed); 9) sample size; 10) psychotic disorders diagnoses (i.e., schizophrenia only, schizophrenia spectrum disorders, or any psychotic disorders including psychotic mood disorders); 11) assessment methods (i.e., structured interview, medical diagnosis, other) and diagnostic criteria; 12) assessment time frame (i.e., current or lifetime). The primary outcome measured was the total count of individuals diagnosed with psychotic disorders. Study authors were contacted in cases of missing data. Any disagreements that arose were thoroughly discussed and resolved through consensus or resolved by a third reviewer (L. Béchard).

### Study quality

The evaluation of the risk of bias in the included studies was carried out by the principal investigator (O.C.) and two undergraduate psychology students (E.A., L. Bachand) using the Joanna Briggs Institute (JBI) critical appraisal checklist for systematic reviews of prevalence data [36]. This nine-item checklist is recognized as the most suitable tool for assessing the methodological quality of prevalence studies [37]. The adequacy of the study sample was evaluated based on a hypothesized prevalence of schizophrenia spectrum and other psychotic disorders of 5% in people with PBG and a 95% confidence level [38]. Each study was assigned a quality score ranging from 0 to 9, with 1 point allocated for meeting each criterion. An overall risk of bias was then determined and categorized as high (0–3), moderate (4–6), or low (7–9) risk [36]. These steps were facilitated by the Covidence systematic review software [34]. In instances of disagreements, consensus was reached through comprehensive discussions or resolved by a third reviewer (L. Béchard).

### Statistical analysis

The main characteristics of each study were summarized using descriptive statistics. The prevalence was described using a box plot and outliers identified in this plot were removed in sensitivity analyses. The overall pooled prevalence estimate of schizophrenia spectrum and other psychotic disorders and its corresponding confidence interval (CI) were calculated by pooling the raw prevalence from each included study using a random effects generalized linear mixed model [39]. Further sensitivity analyses were performed on the overall pooled prevalence in random effects models using the inverse variance method and different transformations, namely the Freeman–Tukey double-arcsine transformation and the logit transformation. The presence of heterogeneity was evaluated using a *χ*^2^ test, the between-study variance with *τ*^2^, and the portion of total variation across studies due to heterogeneity with the *I*^2^ statistic. *I*^2^ values ranging from 0 to 25%, 26 to 50%, 51 to 75%, and 76 to 100% indicated low, moderate, substantial, and considerable heterogeneity, respectively [40]. To explore potential sources of heterogeneity, subgroup meta-analyses were conducted for predetermined categorical moderators, including study publication year (before/after classification of gambling disorder as a behavioral addiction in the DSM-5, i.e., 2013), geographic region, country income, type of recruitment, treatment setting, psychotic disorder diagnoses, assessment method and time frame, and risk of bias. The results are visually presented through forest plots. All reported CIs represent the 95% range. All statistical analyses were conducted with R software, version 4.3.0 (R Project for Statistical Computing), using the meta and metafor packages [41, 42]. Analyses were performed by the principal investigator and a biostatician (O.C. and P.H.C.).

## Results

**Figure 1.**
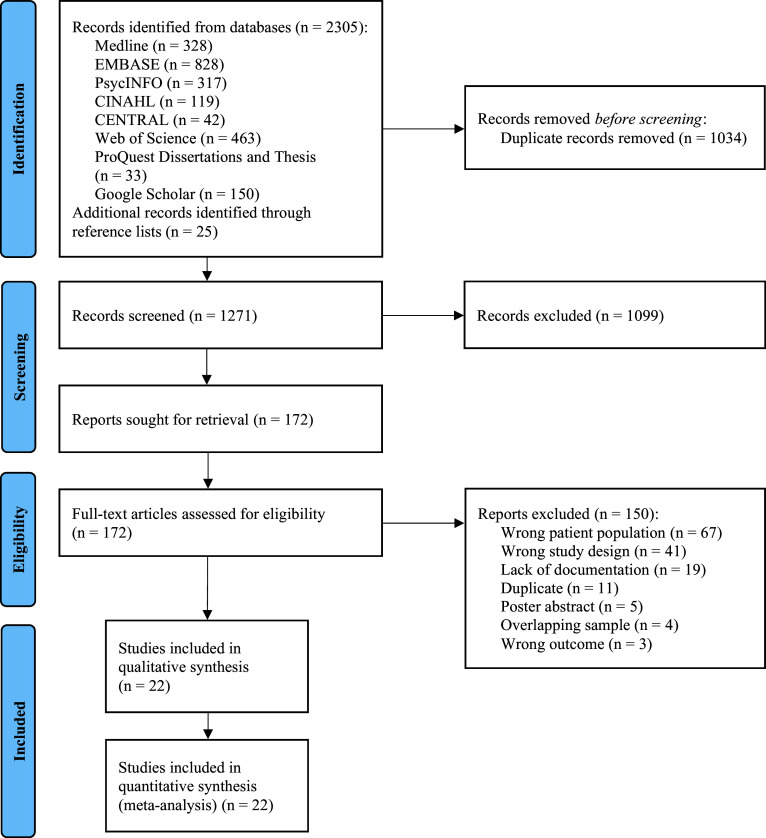
Flow chart of reviewed articles. Adapted from Page et al. [33].

The electronic search yielded a total of 1,271 unique records after the removal of duplicates. Eligibility was assessed for 172 full-text articles, of which 22 non-overlapping studies met the predefined inclusion criteria (Figure 1) [25, 26, 31, 32, 43–60].

### Study characteristics

All 22 studies were included in the meta-analysis, collectively encompassing data from 19,131 individuals with PBG. As shown in Table 1, 12 studies (55%, *n* = 9,884) were conducted in the Americas, six (27%, *n* = 8,599) in Europe, and four (18%, *n* = 648) in the Western Pacific. All but two studies were carried out in high-income countries. Most of the included studies were cross-sectional (8/22; 36%) and cohort studies (7/22; 32%). There were 14 studies (64%, *n* = 6,819) conducted with treatment-seeking problem gamblers, of which six were carried out in outpatient settings, five in inpatient settings, and three in mixed settings. When explicitly documented, psychotic disorders reported were mostly schizophrenia spectrum disorders (13/22; 59%), while five studies focused only on schizophrenia and two included psychotic mood disorders (Table 2). The risk of bias was assessed as low in five studies (23%), moderate in 16 (73%), and high in one study (5%); the average score was 5.4 (range, 3–8; Supplementary Table 2).

**Table 1.** Characteristics of the included studies

Author and year	Country	Design	Type of recruitment	Treatment setting	Risk of bias
Bellaire and Caspari, 1992	Germany	Cross-sectional	Treatment-seeking	Inpatients	Moderate
Grall-Bronnec et al., 2010	France	Cross-sectional	Treatment-seeking	Outpatients	Moderate
Granero et al., 2021	Spain	Cohort	Treatment-seeking	Outpatients	Low
Grubbs et al., 2023	United States	Cross-sectional	Treatment-seeking^a^	Inpatients	Moderate
Jiménez-Murcia et al., 2009	Spain	Cross-sectional	Treatment-seeking	Outpatients	Low
Kausch, 2003	United States	Cohort	Treatment-seeking^a^	Inpatients	Moderate
Kim et al., 2018	Brazil	Cohort	Treatment-seeking	Outpatients	Low
Ladouceur et al., 2006	Canada	Non-randomised experimental	Treatment-seeking	Mixed	Moderate
McCormick et al., 1984	United States	Cohort	Treatment-seeking^a^	Inpatients	Moderate
Pavarin et al., 2018	Italy	Cohort	Treatment-seeking	Mixed	Low
Shek et al., 2013	China	Cohort	Treatment-seeking	Outpatients	Moderate
Specker et al., 1996	United States	Case-control	Treatment-seeking	Outpatients	Moderate
Taber et al., 1987	United States	Cohort	Treatment-seeking^a^	Inpatients	Moderate
Yamada et al., 2023	Japan	Cross-sectional	Treatment-seeking	Mixed	Moderate
Black and Moyer, 1998	United States	Cross-sectional	Survey/recruited	NA	Moderate
Cunningham-Williams et al., 1998	United States	Prevalence/survey	Survey/recruited	NA	Moderate
Grant and Potenza, 2006	United States	Cross-sectional	Survey/recruited	NA	Moderate
Winslow et al., 2010	Singapore	Case-control	Survey/recruited	NA	Moderate
Edens and Rosenheck, 2012	United States	Case-control	Register-based^a^	NA	Moderate
Larsson and Håkansson, 2022	Sweden	Case-control	Register-based	NA	Low
Stefanovics et al., 2023	United States	Cross-sectional	Register-based^a^	NA	Moderate
Dougherty et al., 2020	Australia	Case-control	Other^b^	NA	High

Abbreviations: NA, not applicable.

a United States Armed Forces Veterans.

b Fraud offenders.

**Table 2.** Psychotic disorders assessment in the included studies

Author and year	Sample size (n)	Number of cases (n)	Psychotic disorder diagnoses	Diagnostic criteria	Assessment method	Assessment time frame
Bellaire and Caspari, 1992	51	3	Schizophrenia only	NS	Medical diagnosis	NS
Grall-Bronnec et al., 2010	24	4	NS	DSM-IV	Structured interview	Lifetime
Granero et al., 2021	3,754	166	Schizophrenia only	DSM-IV/5	Medical diagnosis	Lifetime
Grubbs et al., 2023	401	12	Any psychotic disorders^a^	DSM-IV-TR/5	Medical diagnosis	Current
Jiménez-Murcia et al., 2009	498	24	SSD	DSM-IV-TR	Structured interview	Lifetime
Kausch, 2003	113	6	SSD	NS	Medical diagnosis	NS
Kim et al., 2018	349	25	SSD	DSM-IV-TR	Structured interview	Current
Ladouceur et al., 2006	233	27	SSD	DSM-IV	Structured interview	Current
McCormick et al., 1984	50	1	SSD	Other^b^	Structured interview	Current
Pavarin et al., 2018	680	30	SSD	NS	Medical diagnosis	Lifetime
Shek et al., 2013	201	5	SSD	DSM-IV	Structured interview	Lifetime
Specker et al., 1996	40	1	SSD	DSM-III-R	Structured interview	Lifetime
Taber et al., 1987	66	2	NS	NS	Medical diagnosis	Current
Yamada et al., 2023	359	6	Schizophrenia only	DSM-5	Medical diagnosis	NS
Black and Moyer, 1998	30	1	SSD	DSM-III-R	Structured interview	Lifetime
Cunningham-Williams et al., 1998	161	6	Schizophrenia only	DSM-III	Structured interview	Lifetime
Grant and Potenza, 2006	105	0	SSD	DSM-IV	Structured interview	Lifetime
Winslow et al., 2010	40	1	SSD	DSM-III-R/ICD-10	Other^c^	Lifetime
Edens and Rosenheck, 2012	2,283	265	Schizophrenia only	NS	Medical diagnosis	NS
Larsson and Håkansson, 2022	3,592	217	SSD	ICD-10	Medical diagnosis	Lifetime
Stefanovics et al., 2023	6,053	396	SSD	ICD-9/10	Medical diagnosis	Current
Dougherty et al., 2020	48	8	Any psychotic disorders^a^	NS	Medical diagnosis	NS

Abbreviations: DSM, Diagnostic and Statistical Manual of Mental Disorders; ICD, International Classification of Diseases; NS, not specified; SSD, schizophrenia spectrum disorders.

a Schizophrenia spectrum and other psychotic disorders, including psychotic mood disorders.

b Research diagnostic criteria.

c Computerised version of the Composite International Diagnostic Interview (CIDI-Auto).

**Figure 2.**
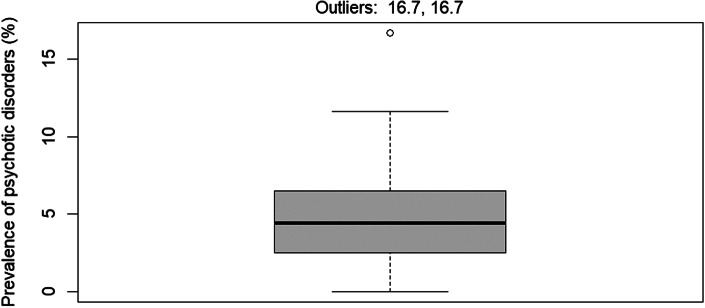
Box plot of the prevalence of any psychotic disorders in people with problem gambling.

The mean age of the populations studied ranged from 33.7 to 53.6 years (Supplementary Table 3), with six studies explicitly stating that only individuals over 18 years of age were included (over 17 years in one study). The proportion of women was 47% in one study, while the proportion of men ranged from 59 to 100% in all the other included studies (over 90% in 11 studies). Of the eight studies that reported ethnicity, the proportion of individuals of White ethnicity ranged from 63 to 97% and that of African/Afro-Americans from 0 to 31%. Three studies were conducted in Asia, of which only one explicitly reported that only Asians were included. The median prevalence of schizophrenia spectrum and other psychotic disorders reported in the included studies was 4.4% (range = 0.0–16.7%, interquartile range = 2.6–6.4%), and two studies were identified as outliers [46, 48], both reporting prevalences of 16.7% (Figure 2).

### Pooled and stratified prevalence of schizophrenia spectrum and other psychotic disorders

**Figure 3.**
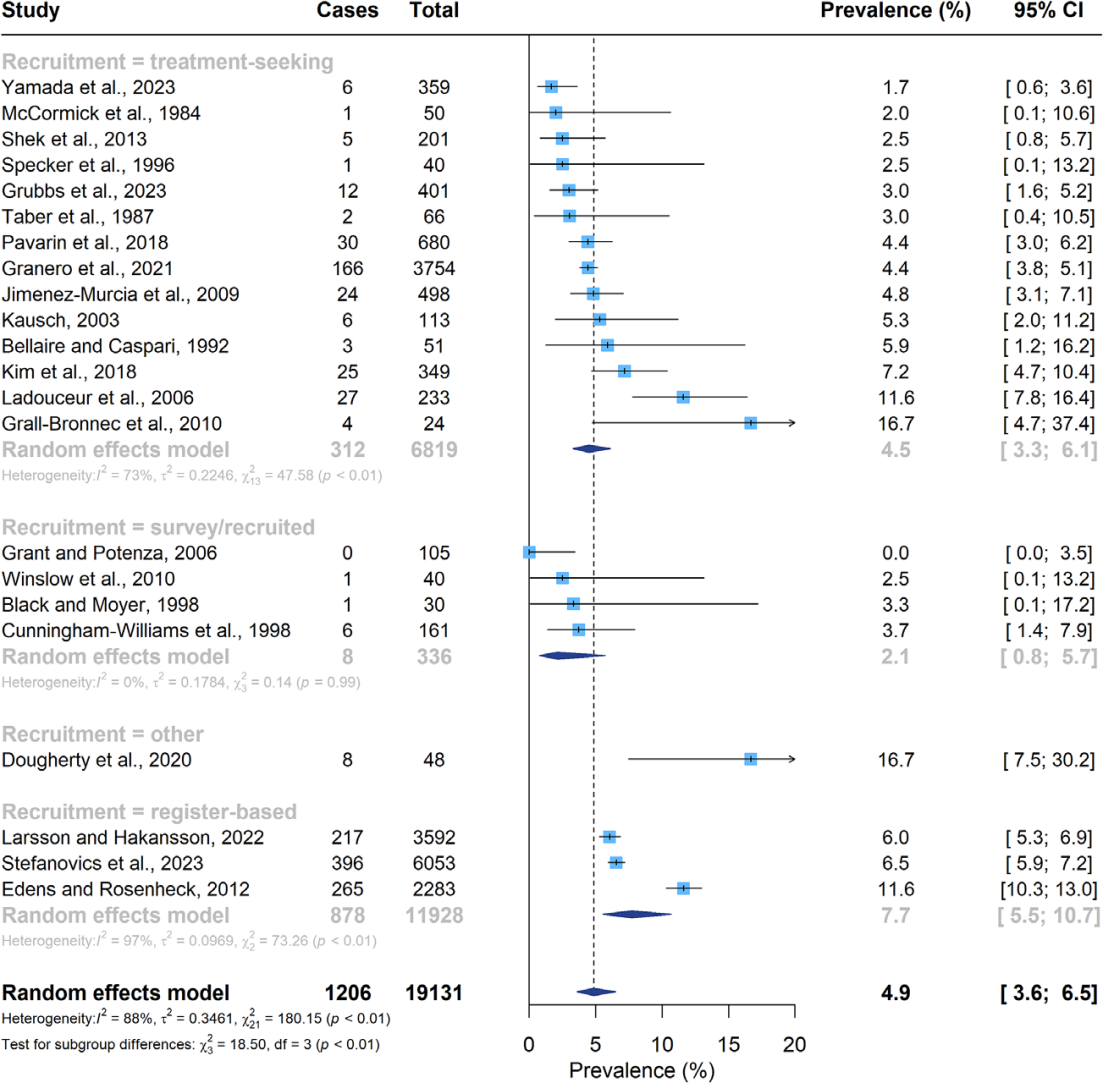
Forest plot of the pooled estimated prevalence of any psychotic disorders in people with problem gambling according to type of recruitment. Abbreviations: CI, confidence interval. *Note:* The overall pooled estimate is represented by the vertical dashed line.

The overall pooled prevalence of schizophrenia spectrum and other psychotic disorders in people with PBG was 4.9% (95% CI, 3.6–6.5%, *I*^2^ = 88%), with considerable heterogeneity. The results of the subgroup analyses are shown in Table 3. There was a subgroup difference (*P* = 0.01) found in the prevalence according to the type of recruitment used in the studies; the prevalence of schizophrenia spectrum and other psychotic disorders was 2.1% (95% CI, 0.8–5.7%, *I*^2^ = 0%) in surveyed/recruited populations, 4.5% (3.3–6.1%, *I*^2^ = 73%) in individuals seeking treatment for PBG, and 7.7% (5.5–10.7%, *I*^2^ = 97%) in register-based studies (Figure 3). Differences in publication year, geographic region, country income, treatment setting, psychotic disorder diagnoses included, method of psychotic disorder assessment, time frame, and risk of bias did not significantly contribute to the heterogeneity found in the overall prevalence of psychotic disorders (Table 3 and Supplementary Figures 1–8). In particular, the prevalence of schizophrenia only was 4.6% (95% CI, 2.5–8.5%, *I*^2^ = 97%), of schizophrenia spectrum disorders 4.9% (95% CI, 3.5–6.8%, *I*^2^ = 55%), and of any psychotic disorders including psychotic mood disorders 6.7% (95% CI, 1.9–20.9%, *I*^2^ = 93%), but no significant differences emerged (*P* = 0.86). Similarly, the current and lifetime prevalences of schizophrenia spectrum and other psychotic disorders were not significantly different (5.6% [95% CI, 3.6–8.7%, *I*^2^ = 75%] and 4.5% [95% CI, 3.5–5.7%, *I*^2^ = 53%], respectively, *P* = 0.39).

**Table 3.** Results of moderator analyses for the prevalence of any psychotic disorders in people with problem gambling

Moderators	Number of studies	Prevalence (95% CI)	*I*^2^ (%)	τ^2^	*P* value^a^
Year of publication					0.68
Before 2013	14	4.5 (2.8–7.1)	76	0.05	
After 2013	8	5.1 (3.6–7.1)	85	0.22	
Geographic region					0.77
Americas	12	4.7 (3.0–7.4)	87	0.40	
Europe	6	5.1 (4.3–6.0)	69	0.02	
Western Pacific	4	3.6 (1.3–9.7)	87	0.87	
Country income					0.91
High	20	4.9 (3.6–6.7)	89	0.37	
Middle	2	4.7 (2.2–9.5)	80	0.19	
Type of recruitment^b^					0.01
Treatment-seeking	14	4.5 (3.3–6.1)	73	0.22	
Survey/recruited	4	2.1 (0.8–5.7)	0	0.18	
Register-based	3	7.7 (5.5–10.7)	97	0.10	
Treatment setting^c^					0.43
Inpatients	5	3.5 (2.4–5.2)	0	0	
Outpatients	6	4.6 (4.1–5.3)	65	0	
Mixed	3	4.6 (1.9–10.9)	92	0.60	
Psychotic disorder diagnoses^d^					0.86
Only schizophrenia	5	4.6 (2.5–8.5)	97	0.42	
Schizophrenia spectrum	13	4.9 (3.5–6.8)	55	0.18	
Any psychotic disorders	2	6.7 (1.9–20.9)	93	0.74	
Psychotic disorder assessment method^e^					0.53
Medical diagnosis	11	5.3 (3.7–7.5)	93	0.29	
Structured interview	10	4.3 (2.4–7.4)	65	0.51	
Assessment time frame^f^					0.39
Current	6	5.6 (3.6–8.7)	75	0.21	
Lifetime	11	4.5 (3.5–5.7)	53	0.04	
Risk of bias^g^					0.32
Low	5	5.2 (4.4–6.1)	70	0.02	
Moderate	16	4.1 (2.7–6.3)	87	0.51	

a For subgroup differences (random effects).

b Other type of recruitment (i.e., fraud offenders chart review) in 1 study.

c In studies conducted among treatment-seeking patients (14 studies).

d Not specified for 2 studies.

e Computer-assisted version of the Composite International Diagnostic Interview (CIDI-Auto) for 1 study.

f Not specified for 5 studies.

g High risk of bias for 1 study.

### Sensitivity analyses

Removal of the two studies identified as outliers resulted in an overall pooled prevalence of schizophrenia spectrum and other psychotic disorders of 4.5% (95% CI, 3.3–6.0%, *I*^2^ = 89%; Supplementary Figure 9). Further sensitivity analyses were performed using the inverse variance method. In a random effects model using the Freeman–Tukey double-arcsine transformation, the pooled prevalence of schizophrenia spectrum and other psychotic disorders was 4.8% (95% CI, 3.4–6.4%, *I*^2^ = 89%; Supplementary Figure 10), with considerable heterogeneity. The overall prevalence using a random effects model and the logit transformation was 5.5% (95% CI, 4.2–7.2%, *I*^2^ = 89%; Supplementary Figure 11), with considerable heterogeneity. The latter method resulted in the exclusion of one study, which reported a 0% prevalence of schizophrenia spectrum and other psychotic disorders in 105 problem gamblers [49].

## Discussion

This study identified an estimated prevalence of psychotic disorders of 4.9% among 19,131 individuals with PBG from a variety of countries (from the Americas, Europe, and Western Pacific regions), different types of recruitment (treatment-seeking individuals, surveys, population-wide registers), and treatment settings (inpatients, outpatients, mixed). Of the factors examined, only the type of recruitment significantly contributed to the heterogeneity found. Noteworthy, the prevalence of psychotic disorders among individuals seeking treatment for PBG, who may be more representative of people encountered in clinical settings, was 4.5%; this is consistent with both the overall estimated prevalence and that of 4.7% found in a previous review [13], albeit in a total sample almost 20 times larger.

Although the prevalence of psychotic disorders in people with PBG may seem lower than that reported for other conditions, such as anxiety and mood disorders, their relative prevalence in the general population must be considered [16]. A recent meta-analysis estimated the global lifetime prevalence of schizophrenia spectrum and other psychotic disorders in the general population to be 1.0% (95% CI, 0.7–1.2%, 28 studies) [30], and nearly 24 million people worldwide were estimated to be affected by schizophrenia in 2019 [20]. In contrast, global lifetime prevalence estimates for mood and anxiety disorders have been reported to be 9.6% (95% CI, 8.5–10.7%, 83 studies) and 12.9% (95% CI, 11.3–14.7%, 70 studies), respectively [61]. Furthermore, although the global prevalence of schizophrenia is relatively low, it is amongst the most invalidating disorders worldwide in terms of quality of life, disability, mortality, and societal costs, which is especially concerning in low- and middle-income countries [18–20]. In addition to evidence suggesting that psychotic disorders in PBG may lead to more severe gambling problems and increased suicidality [21–27], PBG in psychotic disorders may, in turn, be associated with symptom exacerbation, greater symptom severity and psychological distress, and an increased likelihood of suffering from other comorbid mental health disorders, particularly substance abuse [31, 53, 59, 62, 63]. Taken together, these elements warrant greater attention to this comorbidity. To this end, the finding of a lower prevalence of schizophrenia spectrum and other psychotic disorders in surveyed or recruited samples of individuals from the general population suggests that studies conducted in such settings may be less well suited to providing a comprehensive understanding of this comorbidity among problem gamblers.

The prevalence of schizophrenia spectrum and other psychotic disorders in people with PBG estimated in this study (4.9%) was not directly compared with that reported in the general population (1%) [20, 30], but it suggests that it may be increased in the former group. Similarly, the results of our recently published meta-analysis suggested an increased prevalence of PBG in people with psychotic disorders (8.7%) compared with that estimated in the general population (1.3%) [28, 29], although these were also not directly compared. While these two findings suggest that this comorbidity may be relatively common, they do not directly allow us to determine whether people with PBG are less or more likely to have comorbid psychotic disorders than people with psychotic disorders are to have comorbid PBG. In particular, the majority of studies included in each review were cross-sectional in design, limiting the possibility of confirming which of the two conditions preceded the other. While the available literature does not suggest that psychotic disorders per se are the cause of PBG, and vice versa, this comorbidity may be better explained by common risk factors and transition facilitators, including both at the genetic level (e.g., a polygenic risk score for schizophrenia has been associated with increased odds of disordered gambling [64]) and at the personality level (e.g., novelty seeking, reward dependence, and self-transcendence have been found to be central features in patients with schizophrenia and PBG [65]). Although more research is needed to better understand the complex mechanisms involved in the development of this comorbidity, in the meantime, the results of this review inform stakeholders about a clinically relevant issue that needs to be considered in a holistic and recovery-oriented treatment approach.

The included studies were conducted with a wide range of populations, including but not limited to people who have sought treatment for PBG, and from multiple countries and different clinical settings; therefore, the estimated prevalence is more likely to reflect a broader range of the population as a whole, although there are caveats that are described below. This meta-analysis was also carried out according to the highest standards of systematic review and meta-analysis methodology, including pre-planned analyses and a pre-registered study protocol. While the reliability and precision of the results are highly contingent on the quality of the included studies, the risk of bias assessment conducted in this review indicated that the majority of the included studies had a moderate to low risk of bias, which may suggest that the prevalence calculated in this meta-analysis can be considered a good estimate of the true prevalence of schizophrenia spectrum and other psychotic disorders in this population. In this respect, the hypothesis that this prevalence is an underestimate rather than an overestimate is more plausible, as people with a severe mental disorder such as schizophrenia may be less likely to undergo screening for this comorbidity and to seek help for comorbid PBG [66–70].

Of note, four studies included in this review reported prevalences that differed more widely from the estimated overall pooled prevalence. These included two outliers, both of which reported a prevalence of psychotic disorders of 16.7% [46, 48]. Dougherty et al. (2021) examined a sample of 48 individuals who had committed fraud offenses to fund gambling in a study that was judged to be at high risk of bias, mainly due to the lack of a validated method for identifying PBG [46]. Grall-Bronnec et al. (2010), although in a well-conducted study with a low risk of bias, included a limited sample size of 24 problem gamblers, resulting in a very wide confidence interval [48]. Perhaps more interesting are the studies by Edens and Rosenheck (2012) and Ladouceur et al. (2006), which both found a prevalence of psychotic disorders of 11.6% [47, 54]. The first study was conducted in a population-based registry that included data from 1,102,846 United States Armed Forces Veterans who used specialty mental health services in 2009 [47]. The prevalence of psychotic disorders found among the 2,283 individuals with a diagnosis of pathological gambling is, therefore, likely to be overestimated due to the base cohort from which these problem gamblers were identified. This would also help to explain the remaining heterogeneity found in the subgroup of studies conducted using population-based registries, as the other two studies with a similar type of recruitment did not use registries specific to mental health users [26, 32]. In the second study, Ladouceur et al. (2006) examined the prevalence of mental health disorders in a total sample of 233 individuals with a DSM-IV diagnosis of pathological gambling who sought specialized treatment for PBG and voluntarily chose the type of treatment they would receive, i.e., inpatient or outpatient [54]. Interestingly, the prevalence of psychotic disorders differed by type of treatment setting: 4.0% (4/99) among outpatients and 17.2% (23/134) among inpatients, with the most significant difference being for schizoaffective disorders (1.0% versus 9.7%, *P* < 0.01). Again, this may indicate an increased likelihood of comorbid mental health disorders in the base population rather than due to the presence of PBG alone.

The results obtained must also be interpreted considering a few limitations. First, there was considerable heterogeneity in the prevalence estimates obtained in this meta-analysis that could not be explained by the various factors examined, including geographic region, treatment setting, and assessment methods. In particular, and as discussed above, some studies may have had a more significant impact on the overall heterogeneity found due to unique study population (i.e., United States Armed Forces Veterans who had used mental health specialty services) [47] or study design (i.e., experimental study) [54]. Additionally, the limited number of studies in most of the subgroups examined may have prevented the ability to detect significant differences. The prevalence of schizophrenia spectrum and other psychotic disorders in people with PBG may also be expected to vary according to factors not measured in this review, including cultural and social factors. As speculated above, it is possible that people with both a psychotic disorder and PBG are less likely to seek treatment. While the results cannot confirm or refute this hypothesis, the prevalence of schizophrenia spectrum and other psychotic disorders was lowest in surveyed/recruited samples of individuals, followed by treatment-seeking problem gamblers and registry-based populations, suggesting that prevalence may indeed be influenced by the type of recruitment that is used, as well as the base population in which it is measured, as previously discussed.

Second, despite a comprehensive search strategy, there remains a small possibility that some studies reporting on aggregate mental health conditions in individuals with PBG may have been missed, although efforts were made to limit this by manually searching the reference lists of all included studies. Similarly, publication bias is not readily assessable in reviews of prevalence studies [71], so its presence cannot be excluded, although the fact that the risk of bias of the included studies did not have a significant effect on the results, and that both smaller and larger studies reported very low and high prevalence estimates, may indicate only a small effect.

Third, assessment of PBG severity was rare in the included studies, so this information could not be extracted, and the nature and extent of its impact on psychotic disorders and vice versa could not be explored. Likewise, medication use was rarely, if ever, documented in the included studies, again preventing exploration of potential associations with PBG that have been described but remain to be better studied, such as the use of third-generation antipsychotics (i.e., partial dopamine agonists) [72]. Indeed, although mostly documented with the use of aripiprazole [4, 73–79], the occurrence of PBG and other impulse control disorders, such as hypersexuality, has also been reported with the use of brexpiprazole and cariprazine [80, 81], suggesting that their partial dopamine agonist activity may be involved [82]. More specifically, such behavioral adverse events have previously been documented with the use of dopamine agonist drugs in the treatment of Parkinson’s disease, and agonism at central dopamine D3 receptors, in particular, has been suggested as a potential key mediator in this relationship [83]. However, the evidence for an association between third-generation antipsychotics and PBG is mostly limited to case series and analyses of pharmacovigilance databases, which precludes any firm conclusions about causality.

Finally, the results obtained, although from a wide range of geographic regions, types of recruitment, and treatment settings, were still derived from samples composed mainly of White men aged 35 to 50 years living in high-income countries. Further studies with more vulnerable and marginalized populations, particularly in low- and middle-income countries, are therefore needed to gain a better understanding of the complex association between PBG and psychotic disorders, and to explore the consequences for the individual and the implications for preventive and therapeutic approaches.

### Implications

Although relatively infrequent, our findings highlight the relevance of screening problem gamblers for schizophrenia spectrum and other psychotic disorders, as well as any other comorbid mental health conditions, given the important impact such comorbidities can have on the person. When comorbid with psychotic disorders, a holistic and global approach is needed that addresses not only PBG, but also its bidirectional relationship with psychosis. While more research is needed to better understand how this comorbidity varies according to individual and socioeconomic factors, efforts should be made to sensitize clinicians and improve access to health care for these individuals.

## Supporting information

Corbeil et al. supplementary material Corbeil et al. supplementary material

## Data Availability

Template data collection forms, raw data extracted from included studies and used for all analyses are available from the corresponding author O.C. upon reasonable request.

## Supplementary material

The supplementary material for this article can be found at http://doi.org/10.1192/j.eurpsy.2024.1777.
