# Supplementary material for: Prevalence of schizophrenia spectrum and other psychotic disorders in problem gambling: A systematic review and meta-analysis
Source: Eur Psychiatry. 2024 Sep 25;67(1):e56. doi: 10.1192/j.eurpsy.2024.1777 (PMC11536207; doi:10.1192/j.eurpsy.2024.1777)
Supplement: Corbeil et al. supplementary material [file S0924933824017772sup001.docx]

**Supplementary Table 1. Search strategy**

| **Medline (using Ovid)** | |
| --- | --- |
| **#** | **Searches** |
| 1 | gambling/ |
| 2 | (gambl* or betting*).ab,kf,ti. |
| 3 | 1 or 2 |
| 4 | exp psychotic disorders/ or exp schizophrenia/ or affective disorders, psychotic/ or capgras syndrome/ or paranoid disorders/ |
| 5 | (((psychotic* or schizoaffective* or schizotypal* or delusional*) adj5 disorder*) or psychose* or psychosis or schizophreni* or hallucinosis or "capgras syndrome*" or encephalopschychosis or psychoticism* or paranoi* or hebephreni*).ab,kf,ti. |
| 6 | 4 or 5 |
| 7 | 3 and 6 |
| **EMBASE** | |
| **#** | **Searches** |
| 1 | 'gambling'/de OR 'pathological gambling'/de |
| 2 | gambl*:ti,ab,kw OR betting*:ti,ab,kw |
| 3 | #1 OR #2 |
| 4 | 'psychosis'/de OR 'alcohol psychosis'/de OR 'affective psychosis'/de OR 'paranoid psychosis'/exp OR 'schizophrenia'/exp OR 'shared psychotic disorder'/de OR 'Capgras syndrome'/de |
| 5 | (((psychotic* or schizoaffective* or schizotypal* or delusional*) NEAR/5 disorder*) or psychose* or psychosis or schizophreni* or hallucinosis or "capgras syndrome*" or encephalopschychosis or psychoticism* or paranoi* or hebephreni*):ti,ab,kw |
| 6 | #4 OR #5 |
| 7 | #3 AND #6 |
| **PsycINFO (using Ovid)** | |
| **#** | **Searches** |
| 1 | gambling/ OR gambling disorder/ |
| 2 | (gambl* or betting*).ab,ti. |
| 3 | 1 or 2 |
| 4 | psychosis/ or affective psychosis/ or alcoholic psychosis/ or toxic psychoses/ or capgras syndrome/ or exp "paranoia (psychosis)"/ or exp schizophrenia/ |
| 5 | (((psychotic* or schizoaffective* or schizotypal* or delusional*) adj5 disorder*) or psychose* or psychosis or schizophreni* or hallucinosis or "capgras syndrome*" or encephalopschychosis or psychoticism* or paranoi* or hebephreni*).ab,ti. |
| 6 | 4 or 5 |
| 7 | 3 and 6 |
| **CINAHL** | |
| **#** | **Searches** |
| S1 | (MH "Gambling") |
| S2 | TI(gambl* or betting*) |
| S3 | AB(gambl* or betting*) |
| S4 | S1 or S2 or S3 |
| S5 | (MH "Schizophrenia+") OR (MH "Psychotic Disorders") OR (MH "Affective Disorders, Psychotic") OR (MH "Capgras Syndrome") OR (MH "Paranoid Disorders") OR (MH "Psychoses, Substance-Induced+") |
| S6 | TI (((psychotic* or schizoaffective* or schizotypal* or delusional*) N5 disorder*) or psychose* or psychosis or schizophreni* or hallucinosis or "capgras syndrome*" or encephalopschychosis or psychoticism* or paranoi* or hebephreni*) |
| S7 | AB (((psychotic* or schizoaffective* or schizotypal* or delusional*) N5 disorder*) or psychose* or psychosis or schizophreni* or hallucinosis or "capgras syndrome*" or encephalopschychosis or psychoticism* or paranoi* or hebephreni*) |
| S8 | S5 or S6 or S7 |
| S9 | S4 and S8 |
| **CENTRAL** | |
| **#** | **Searches** |
| 1 | [mh "gambling"] |
| 2 | (gambl* or betting*):ti,ab,kw |
| 3 | #1 or #2 |
| 4 | [mh "psychotic disorders"] or [mh "schizophrenia"] or [mh "affective disorders, psychotic"] or [mh "capgras syndrome"] or [mh "paranoid disorders"] |
| 5 | (((psychotic* or schizoaffective* or schizotypal* or delusional*) NEAR/5 disorder*) or psychose* or psychosis or schizophreni* or hallucinosis or "capgras syndrome*" or encephalopschychosis or psychoticism* or paranoi* or hebephreni*):ti,ab,kw |
| 6 | #4 or #5 |
| 7 | #3 and #6 |
| **Web of Science** | |
| **#** | **Searches** |
| 1 | ts=(gambl* or betting*) |
| 2 | ts=(((psychotic* or schizoaffective* or schizotypal* or delusional*) NEAR/5 disorder*) or psychose* or psychosis or schizophreni* or hallucinosis or "capgras syndrome*" or encephalopschychosis or psychoticism* or paranoi* or hebephreni*) |
| 3 | #1 and #2 |
| **ProQuest Dissertation and Thesis** | |
| **#** | **Searches** |
| 1 | noft(gambl* OR betting*) |
| 2 | noft((((psychotic* or schizoaffective* or schizotypal* or delusional*) NEAR/5 disorder*) or psychose* or psychosis or schizophreni* or hallucinosis or "capgras syndrome*" or encephalopschychosis or psychoticism* or paranoi* or hebephreni*)) |
| 3 | 1 and 2 |
| **Google Scholar** | |
| **#** | **Searches** |
| 1 | Gambling psychotic |

**Supplementary Table 2. Quality assessment of the included studies**

| **Author and year** | **Sample frame** | **Sampling method** | **Sample size** | **Descrip-tion** | **Sufficient coverage** | **Valid methods** | **Reliable measure-ment** | **Ana-lysis** | **Response rate** | **Total score (0-9)** |
| --- | --- | --- | --- | --- | --- | --- | --- | --- | --- | --- |
| Bellaire and Caspari, 1992 | 1 | 1 | 0 | 1 | 1 | 0 | 0 | 0 | NA | 4 |
| Black and Moyer, 1998 | 0 | 1 | 0 | 1 | 0 | 1 | 1 | 1 | 0 | 5 |
| Cunningham-Williams et al., 1998 | 0 | 1 | 1 | 1 | 0 | 0 | 0 | 1 | 1 | 5 |
| Dougherty et al., 2020 | 0 | 0 | 0 | 1 | 1 | 0 | 0 | 1 | NA | 3 |
| Edens and Rosenheck, 2012 | 0 | 1 | 1 | 1 | 1 | 1 | 0 | 1 | NA | 6 |
| Grall-Bronnec et al., 2010 | 1 | 1 | 0 | 1 | 0 | 1 | 0 | 1 | 1 | 6 |
| Granero et al., 2021 | 1 | 1 | 1 | 1 | 1 | 0 | 1 | 1 | 1 | 8 |
| Grant and Potenza, 2006 | 1 | 0 | 1 | 0 | 0 | 1 | 1 | 1 | NA | 5 |
| Grubbs et al., 2023 | 0 | 1 | 1 | 1 | 1 | 0 | 0 | 1 | NA | 5 |
| Jiménez-Murcia et al., 2009 | 1 | 1 | 1 | 1 | 1 | 1 | 0 | 1 | NA | 7 |
| Kausch, 2003 | 0 | 1 | 1 | 1 | 1 | 1 | 0 | 1 | NA | 6 |
| Kim et al., 2018 | 1 | 1 | 1 | 1 | 0 | 0 | 1 | 1 | 1 | 7 |
| Ladouceur et al., 2006 | 1 | 1 | 1 | 1 | 0 | 0 | 1 | 1 | 0 | 6 |
| Larsson and Håkansson, 2022 | 1 | 1 | 1 | 1 | 1 | 1 | 0 | 1 | NA | 7 |
| McCormick et al., 1984 | 0 | 1 | 0 | 1 | 1 | 1 | 0 | 0 | NA | 4 |
| Pavarin et al., 2018 | 1 | 1 | 1 | 1 | 1 | 1 | 0 | 1 | NA | 7 |
| Shek et al., 2013 | 1 | 1 | 1 | 1 | 0 | 1 | 0 | 1 | 0 | 6 |
| Specker et al., 1996 | 1 | 1 | 0 | 1 | 0 | 1 | 0 | 1 | 0 | 5 |
| Stefanovics et al., 2023 | 0 | 1 | 1 | 1 | 1 | 0 | 1 | 1 | NA | 6 |
| Taber et al., 1987 | 0 | 1 | 0 | 1 | 1 | 0 | 1 | 0 | NA | 4 |
| Winslow et al., 2010 | 0 | 0 | 0 | 1 | 1 | 1 | 0 | 1 | 0 | 4 |
| Yamada et al., 2023 | 0 | 1 | 1 | 1 | 1 | 0 | 1 | 1 | NA | 6 |
| *Abbreviation: NA, not applicable.* | | | | | | | | | | |

**Supplementary Table 3. Characteristics of the population in the included studies**

| **Author and year** | **Type of population** | **Mean age ± SD**  (years) | **Men**  (%) | **White**  (%) | **African/Afro-American**  (%) |
| --- | --- | --- | --- | --- | --- |
| Bellaire and Caspari, 1992 | Treatment-seeking | 33.7 ± NS | 100 | NS | NS |
| Grall-Bronnec et al., 2010 | Treatment-seeking | 43.8 ± 10.7 | 79 | NS | NS |
| Granero et al., 2021 | Treatment-seeking | 42.0 ± 13.5 | 91 | NS | NS |
| Grubbs et al., 2023 | Treatment-seeking^a^ | 53.6 ± 11.4 | 78 | 70 | 21 |
| Jiménez-Murcia et al., 2009 | Treatment-seeking | 41.5 ± 13.1 | 88 | NS | NS |
| Kausch, 2003 | Treatment-seeking^a^ | 48.9 ± 9.7 | 91 | 86 | 11 |
| Kim et al., 2018 | Treatment-seeking | 47.3 ± 12.2 | 59 | 72 | 8 |
| Ladouceur et al., 2006 | Treatment-seeking | 42.8 ± 11.9 | 73 | NS | NS |
| McCormick et al., 1984 | Treatment-seeking^a^ | 42 ± 9 | 100 | NS | NS |
| Pavarin et al., 2018 | Treatment-seeking | 47.4 ± NS | 80 | NS | NS |
| Shek et al., 2013 | Treatment-seeking | median = 41-50 | 91 | NS | NS |
| Specker et al., 1996 | Treatment-seeking | 41.1 ± 10.7 | 63 | 93 | 0 |
| Taber et al., 1987 | Treatment-seeking^a^ | 43 ± NS | 100 | NS | NS |
| Yamada et al., 2023 | Treatment-seeking | median = 37.0 | 91 | NS | NS |
| Black and Moyer, 1998 | Survey/recruited | 44 ± 14 | 77 | NS | NS |
| Cunningham-Williams et al., 1998 | Survey/recruited | 36.5 ± 16.7 | 78 | 69 | 31 |
| Grant and Potenza, 2006 | Survey/recruited | 46.1 ± 11.5 | 100 | 97 | NS |
| Winslow et al., 2010 | Survey/recruited | 38.3 ± 10.0 | 90 | 0 | 0 |
| Edens and Rosenheck, 2012 | Register-based^a^ | NS | 92 | MD | MD |
| Larsson and Håkansson, 2022 | Register-based | 34 to 40 | 78 | NS | NS |
| Stefanovics et al., 2023 | Register-based^a^ | 53 ± 3 | 94 | 63 | 31 |
| Dougherty et al., 2020 | Other^b^ | 39.9 ± 11.7 | 53 | NS | NS |

*Abbreviations: MD, missing data; NA, not applicable; NS, not specified; SD, standard deviation.*

*^a^United States Armed Forces Veterans.*

*^b^Fraud offenders.*


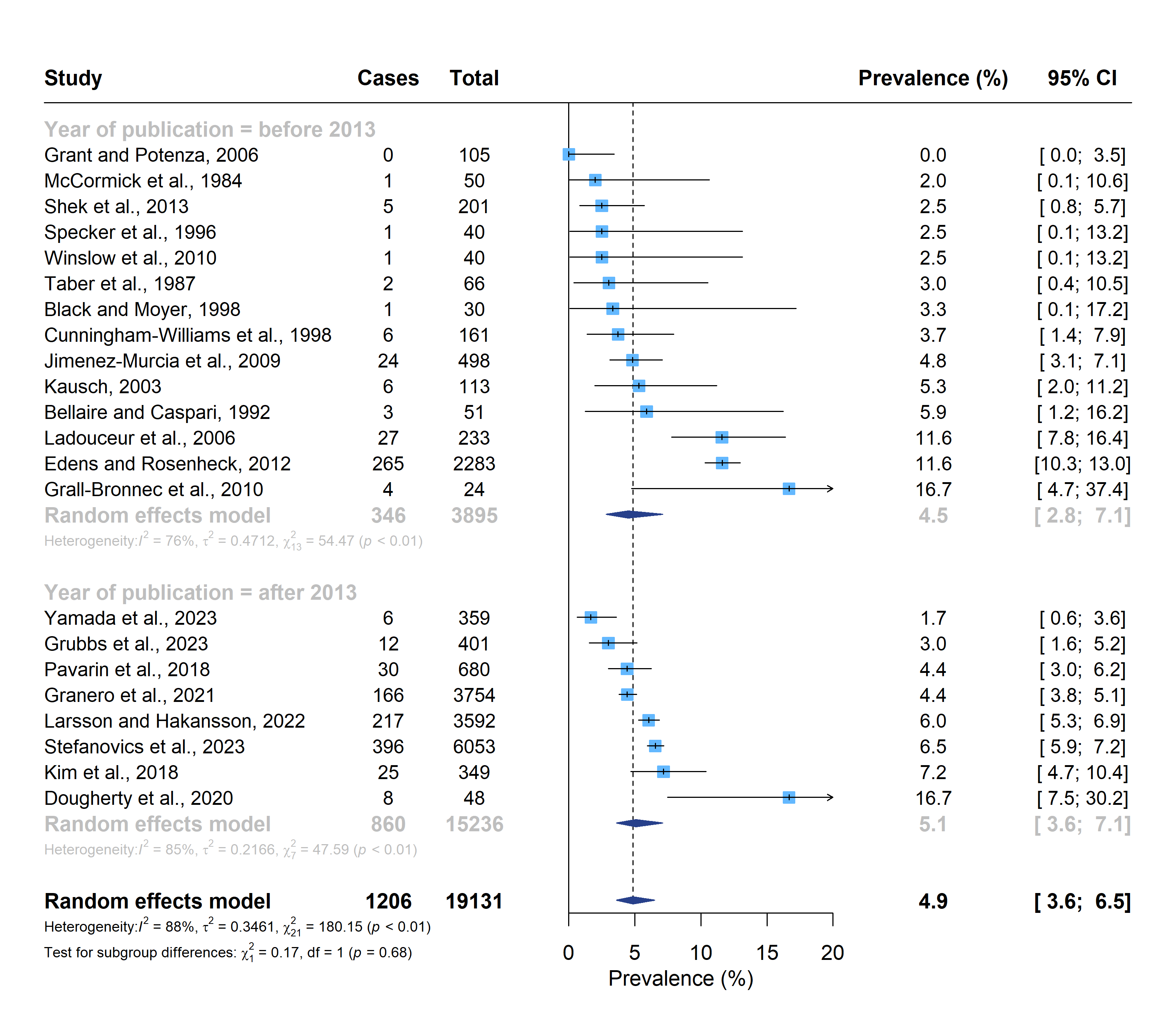


**Supplementary Figure 1. Forest plot of the pooled estimated prevalence of any psychotic disorders in people with problem gambling according to year of publication**

*Abbreviations: CI, confidence interval.*

*Note: The overall pooled estimate is represented by the vertical dashed line.*


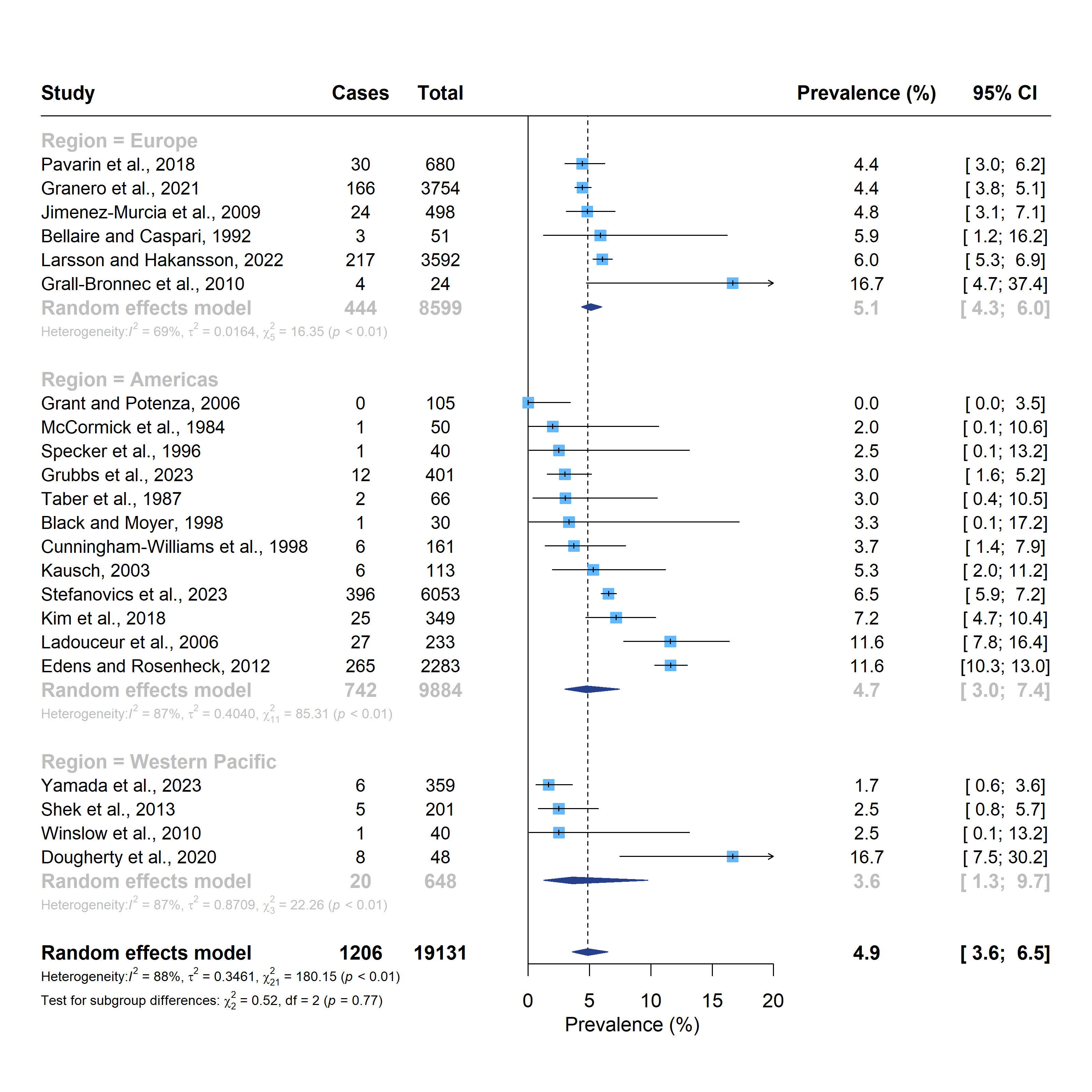


**Supplementary Figure 2. Forest plot of the pooled estimated prevalence of any psychotic disorders in people with problem gambling according to geographic region**

*Abbreviations: CI, confidence interval.*

*Note: The overall pooled estimate is represented by the vertical dashed line.*


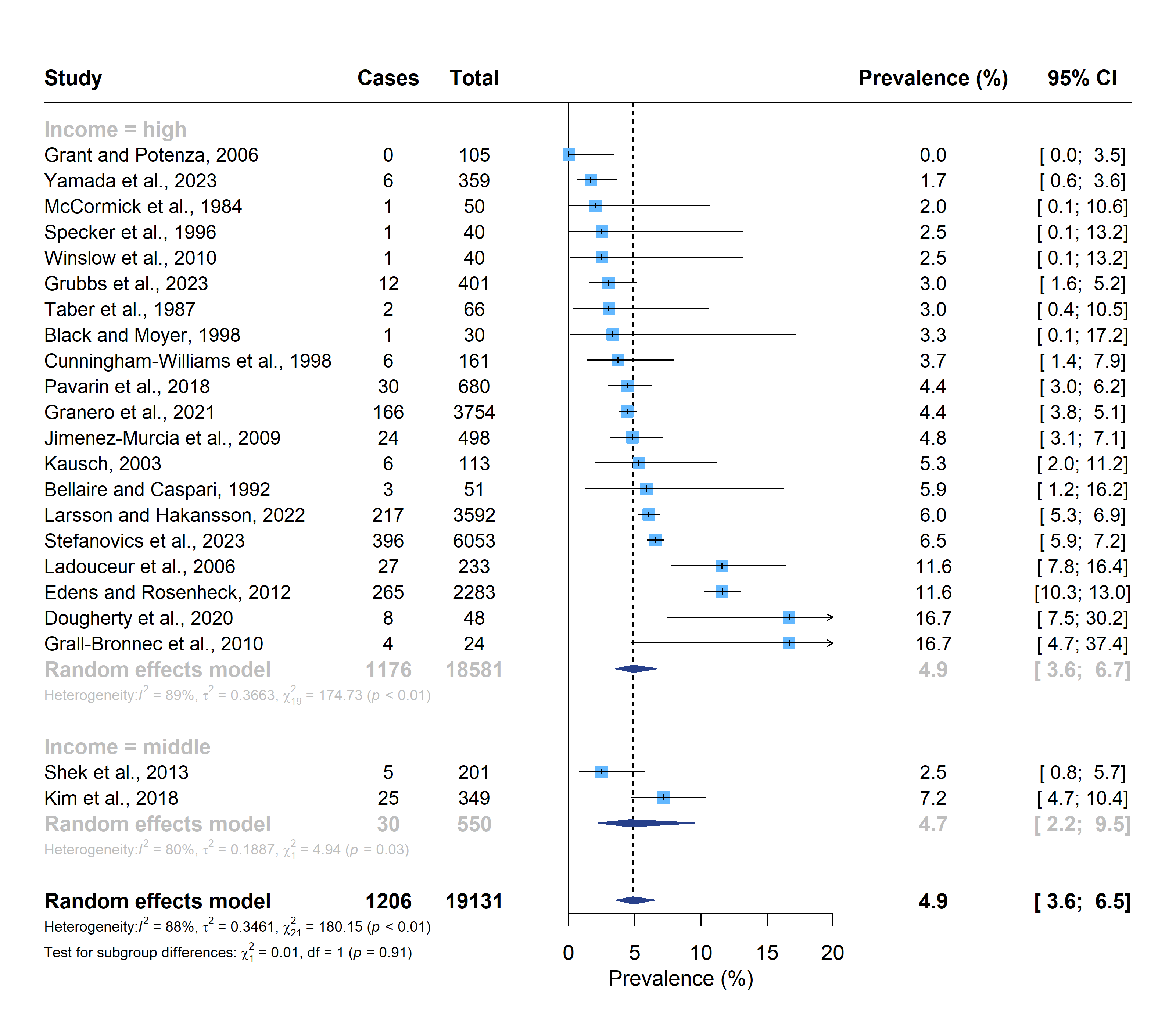


**Supplementary Figure 3. Forest plot of the pooled estimated prevalence of any psychotic disorders in people with problem gambling according to study country income**

*Abbreviations: CI, confidence interval.*

*Note: The overall pooled estimate is represented by the vertical dashed line.*


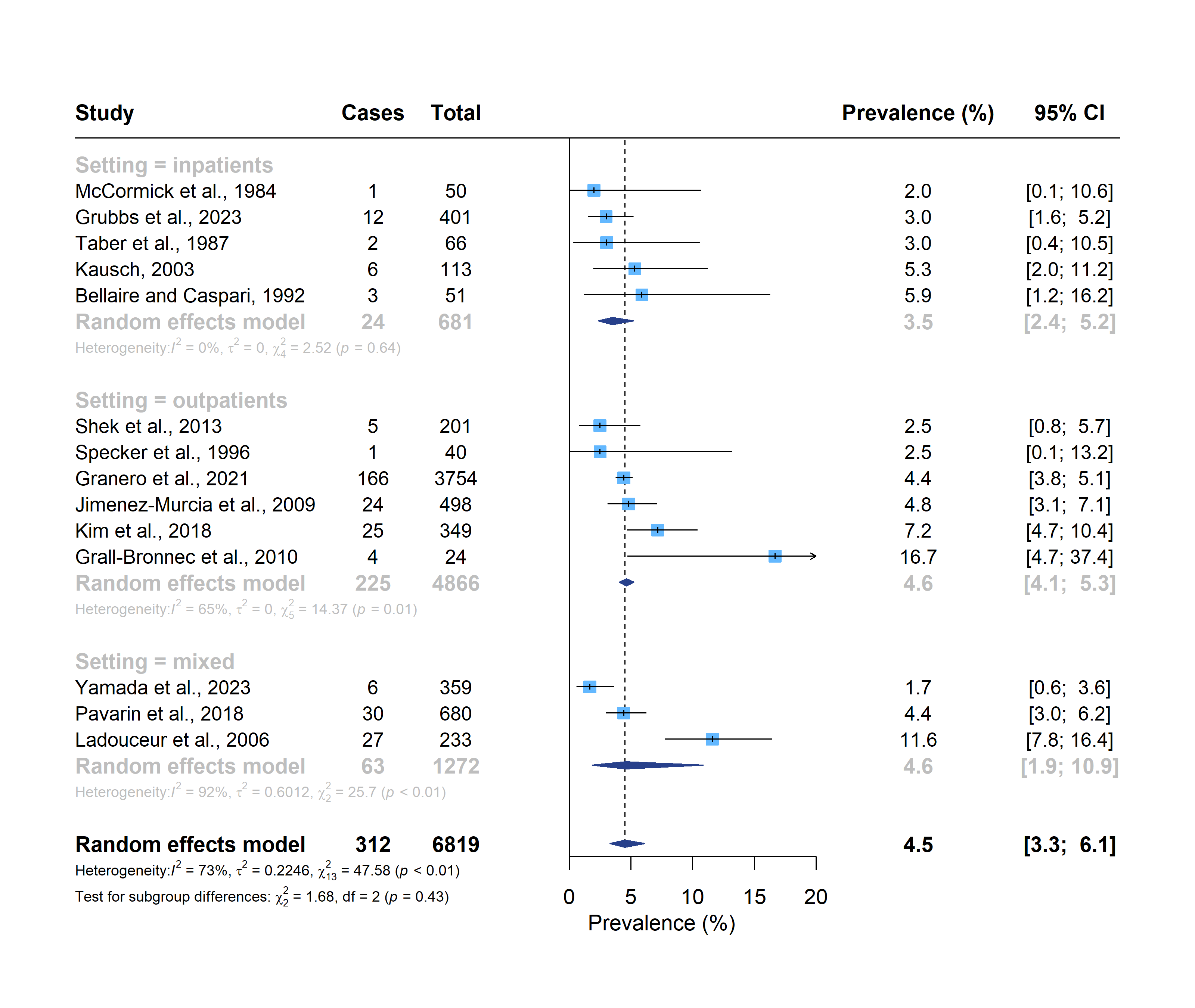


**Supplementary Figure 4. Forest plot of the pooled estimated prevalence of any psychotic disorders in people with problem gambling according to treatment setting**

*Abbreviations: CI, confidence interval.*

*Note: The overall pooled estimate is represented by the vertical dashed line. In studies conducted among treatment-seeking patients (14 studies).*


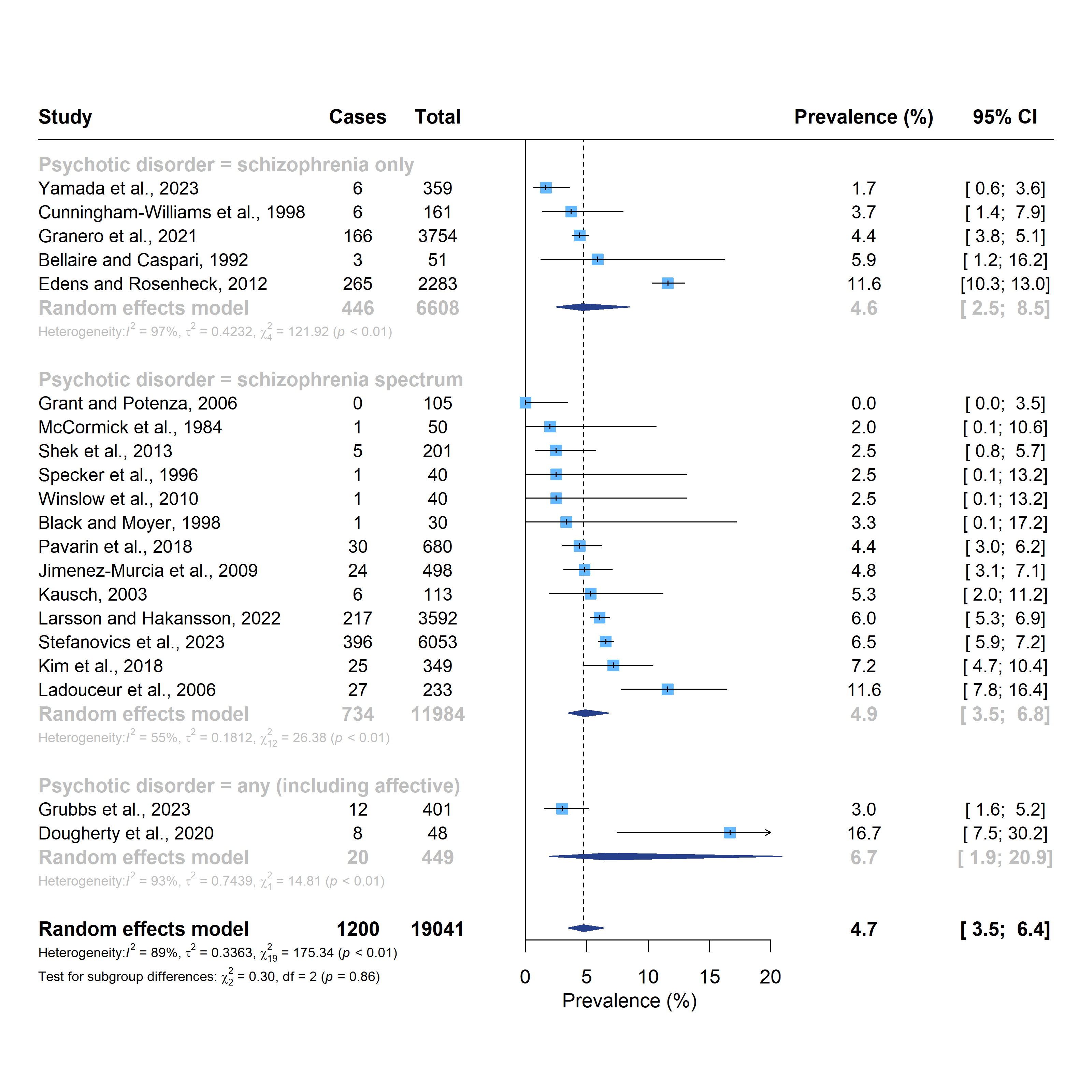


**Supplementary Figure 5. Forest plot of the pooled estimated prevalence of any psychotic disorders in people with problem gambling according to psychotic disorder diagnoses**

*Abbreviations: CI, confidence interval.*

*Note: The overall pooled estimate is represented by the vertical dashed line. Two studies excluded (diagnoses not specified).*


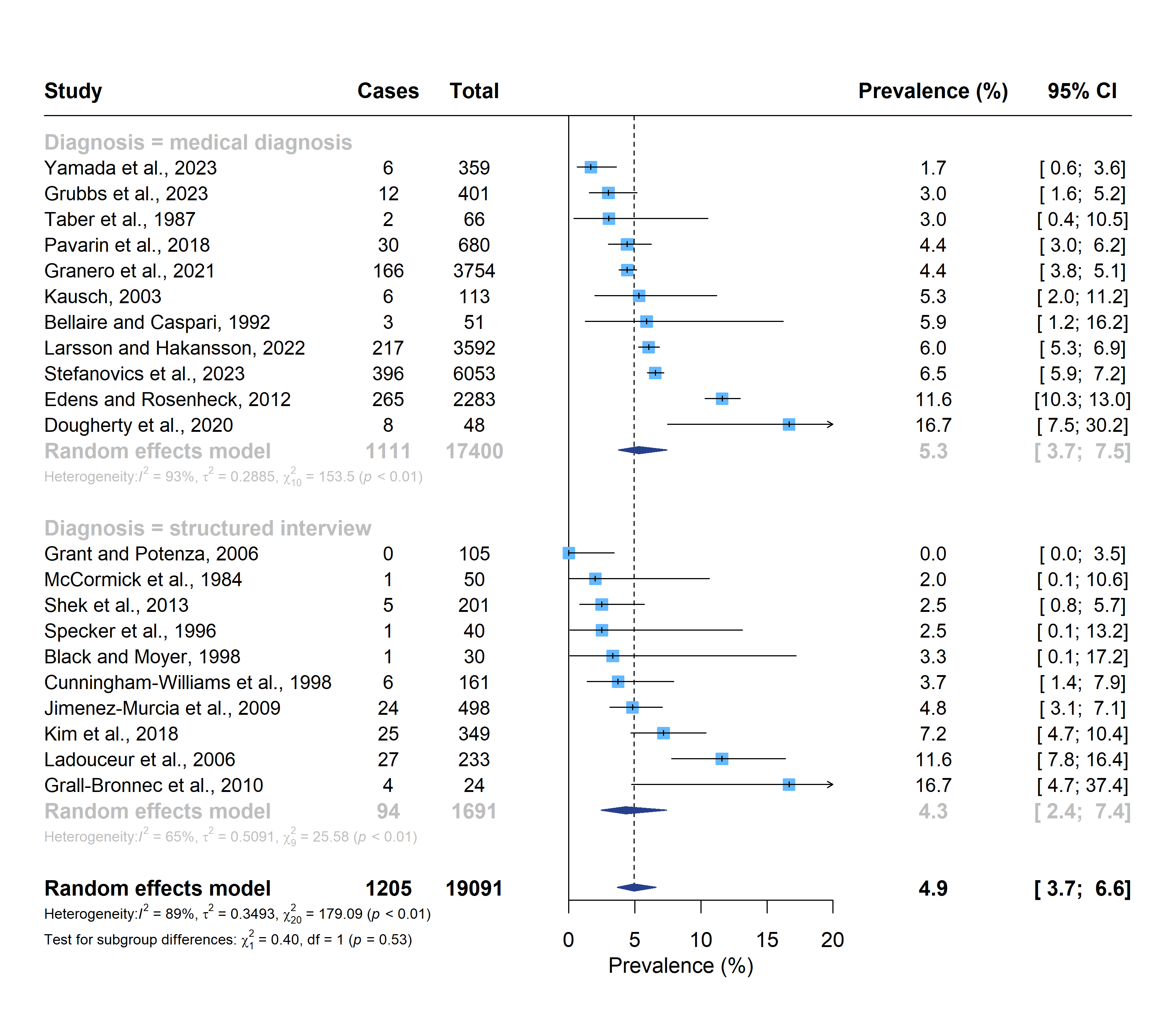


**Supplementary Figure 6. Forest plot of the pooled estimated prevalence of any psychotic disorders in people with problem gambling according to psychotic disorder assessment method**

*Abbreviations: CI, confidence interval.*

*Note: The overall pooled estimate is represented by the vertical dashed line. One study excluded (Computer-assisted version of the Composite International Diagnostic Interview).*


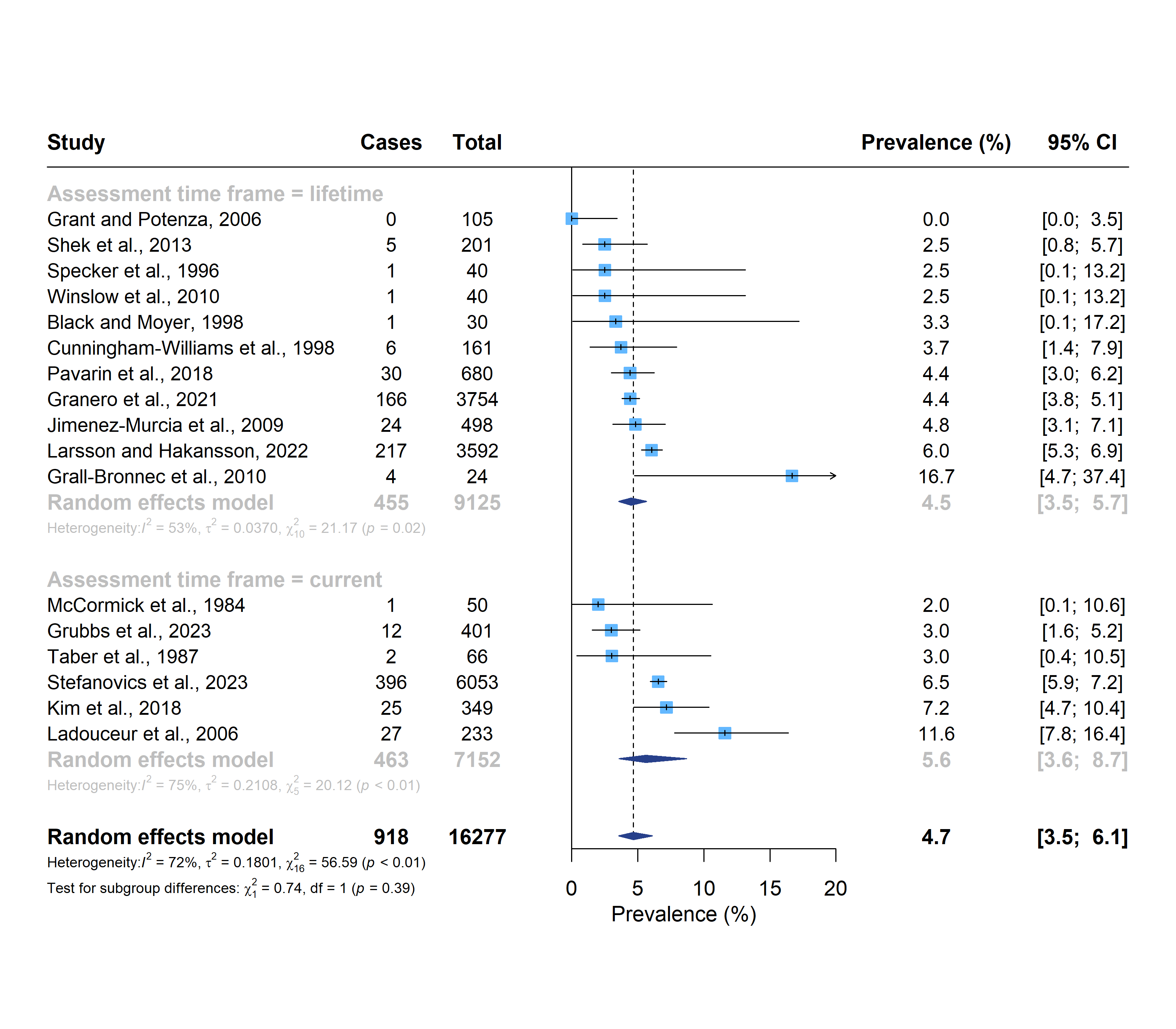


**Supplementary Figure 7. Forest plot of the pooled estimated prevalence of any psychotic disorders in people with problem gambling according to assessment time frame**

*Abbreviations: CI, confidence interval.*

*Note: The overall pooled estimate is represented by the vertical dashed line. Two studies excluded (time frame not specified).*


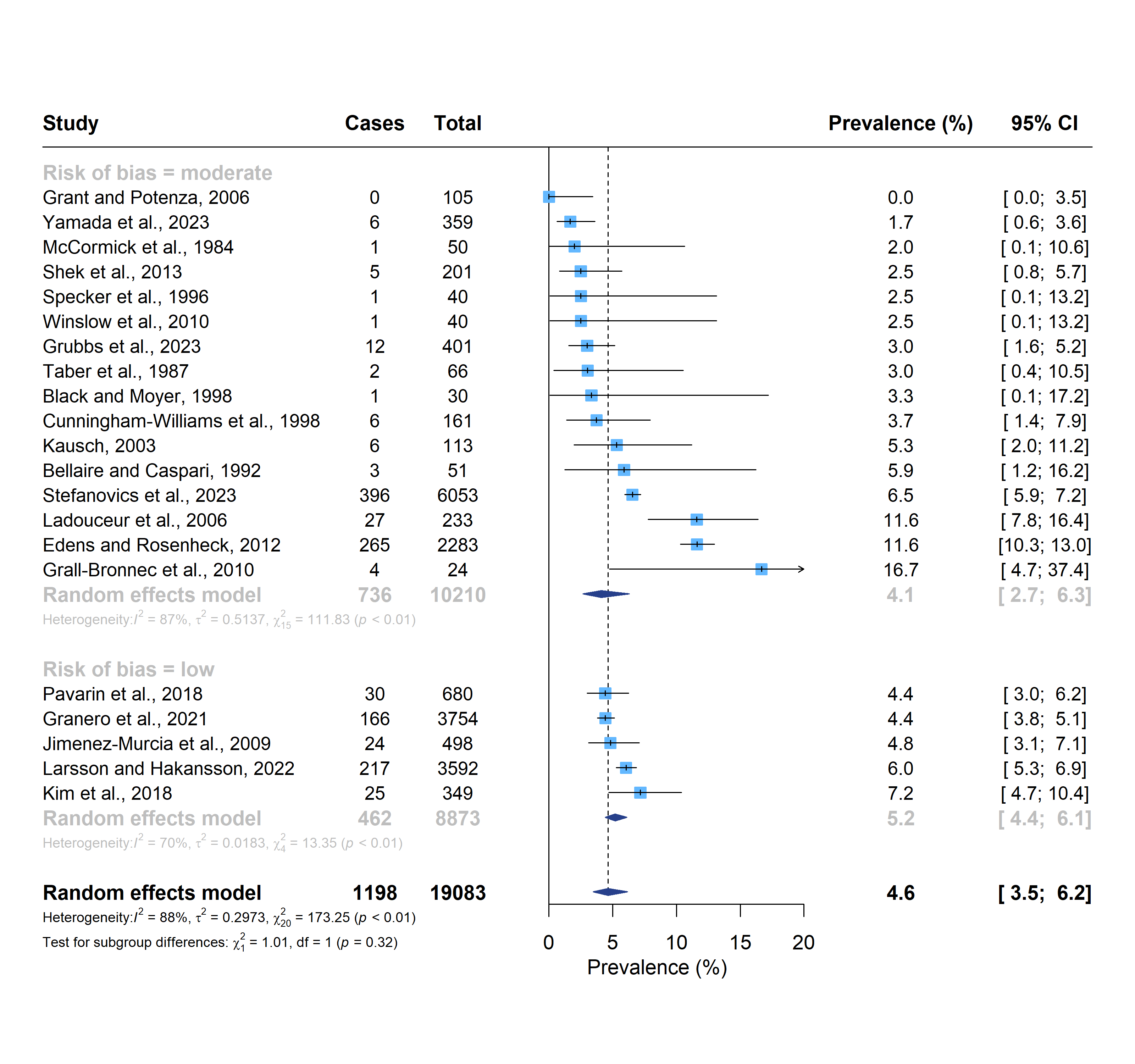


**Supplementary Figure 8. Forest plot of the pooled estimated prevalence of any psychotic disorders in people with problem gambling according to risk of bias**

*Abbreviations: CI, confidence interval.*

*Note: The overall pooled estimate is represented by the vertical dashed line. One study excluded (high risk of bias).*
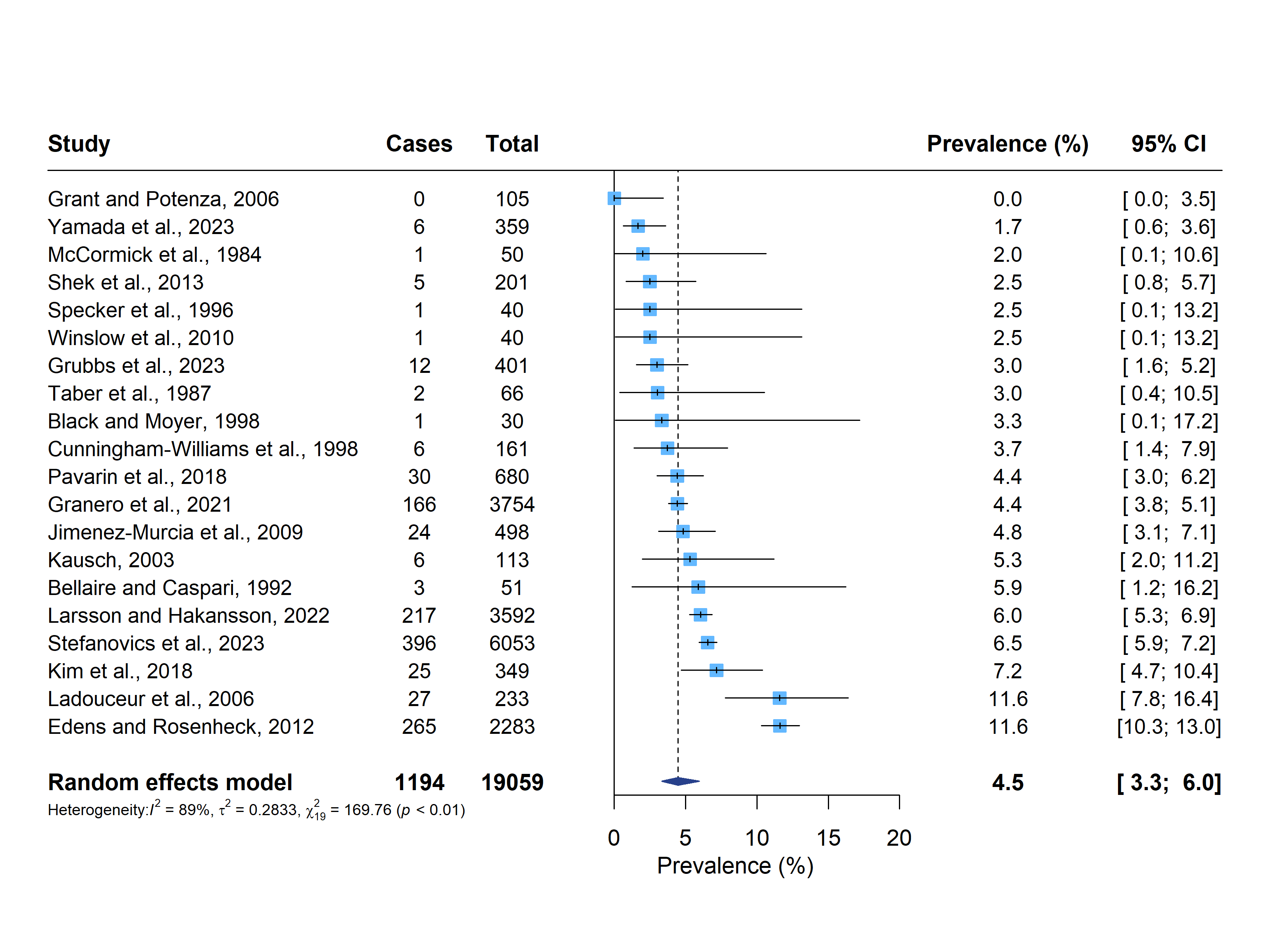
**Supplementary Figure 9. Forest plot of the pooled estimated prevalence of any psychotic disorders in people with problem gambling (excluding two outlier studies)**

*Abbreviations: CI, confidence interval.*

*Note: The overall pooled estimate is represented by the vertical dashed line.*


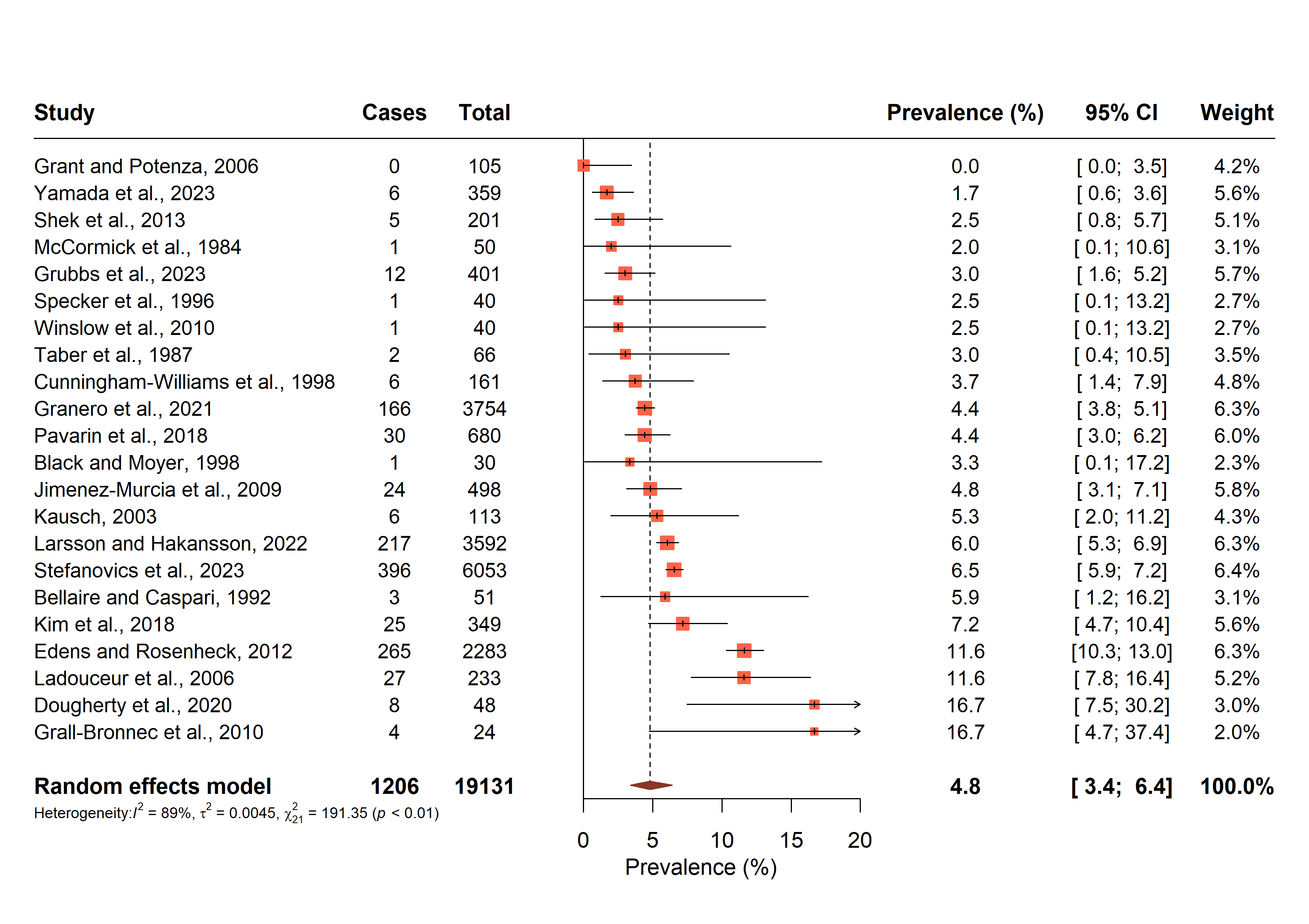


**Supplementary Figure 10. Forest plot of the pooled estimated prevalence of any psychotic disorders in people with problem gambling (inverse-variance method, Freeman-Tukey transformation)**

*Abbreviations: CI, confidence interval.*

*Note: The overall pooled estimate is represented by the vertical dashed line.*
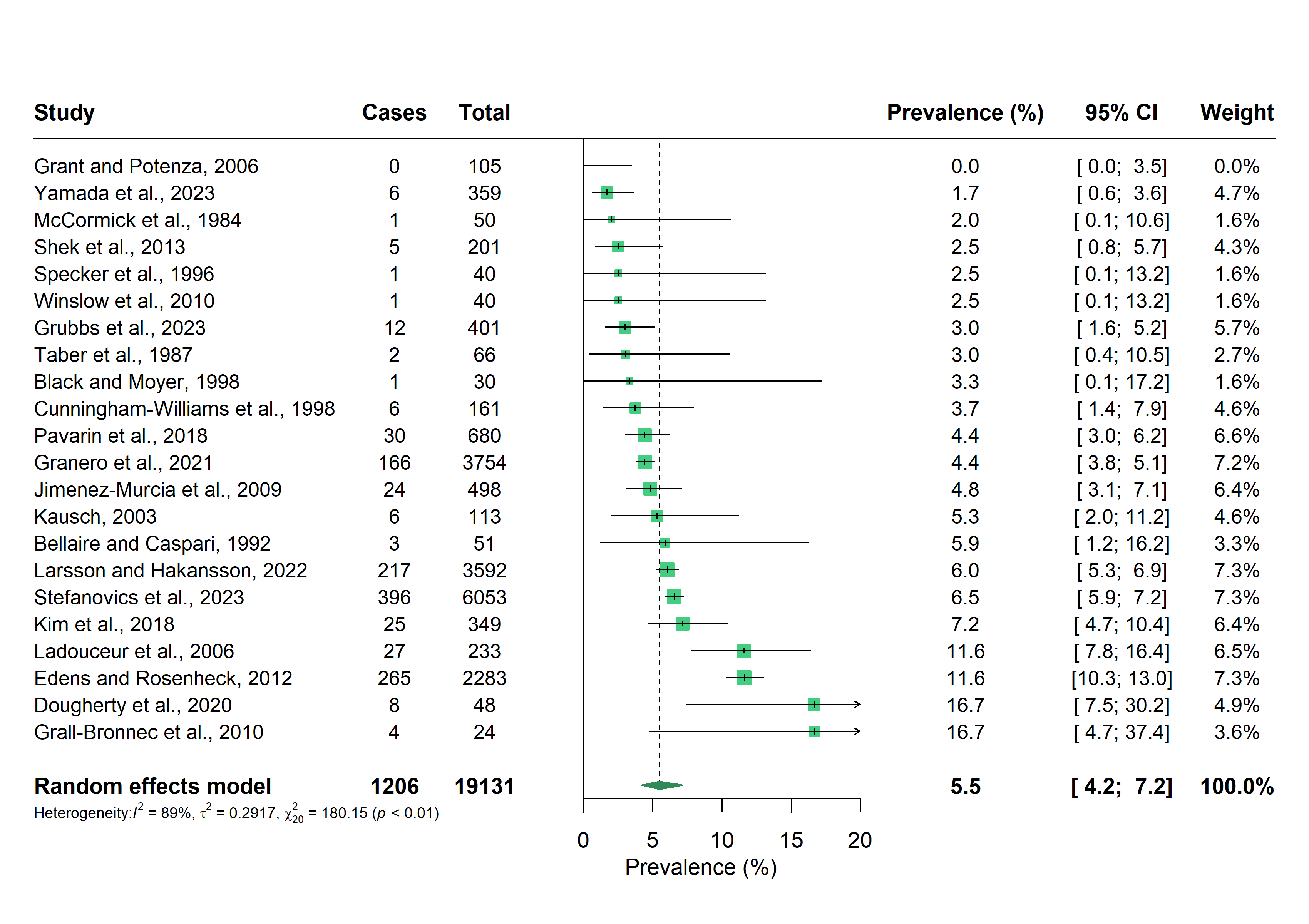


**Supplementary Figure 11. Forest plot of the pooled estimated prevalence of any psychotic disorders in people with problem gambling (inverse-variance method, logit transformation)**

*Abbreviations: CI, confidence interval.*

*Note: The overall pooled estimate is represented by the vertical dashed line.*
